# Assessment of haemorrhoidal artery network using colour duplex imaging and clinical implications

**DOI:** 10.1002/bjs.7700

**Published:** 2011-10-21

**Authors:** C Ratto, A Parello, L Donisi, F Litta, G Zaccone, G B Doglietto

**Affiliations:** Department of Surgical Sciences, Catholic UniversityLargo A. Gemelli, 8, 00168, Rome, Italy

## Abstract

**Background:**

Dearterialization should reduce arterial overflow to haemorrhoids. The purpose of this study was to assess the topography of haemorrhoidal arteries.

**Methods:**

Fifty patients with haemorrhoidal disease were studied. Using endorectal ultrasonography, six sectors were identified within the lower rectal circumference. Starting from the highest level (6 cm above the anorectal junction), the same procedure was repeated every 1 cm until the lowest level was reached (1 cm above the anorectal junction). Colour duplex imaging examinations identified haemorrhoidal arteries related to the rectal wall layers, and the arterial depth was calculated.

**Results:**

Haemorrhoidal arteries were detected in 64·3, 66·0, 66·0, 98·3, 99·3 and 99·7 per cent of the sectors 6, 5, 4, 3, 2 and 1 cm above the anorectal junction respectively (*P* < 0·001). Most of the haemorrhoidal arteries were external to the rectal wall at 6 and 5 cm (97·9 and 90·9 per cent), intramuscular at 4 cm (55·0 per cent), and within the submucosa at 3, 2 and 1 cm above the anorectal junction (67·1, 96·6 and 100 per cent) (*P* < 0·001). The mean arterial depth decreased significantly from 8·3 mm at 6 cm to 1·9 mm at 1 cm above the anorectal junction (*P* < 0·001).

**Conclusion:**

This study demonstrated that the vast majority of haemorrhoidal arteries lie within the rectal submucosa at the lowest 2 cm above the anorectal junction. This should therefore be the best site for performing haemorrhoidal dearterialization.

## Introduction

The anatomical and physiological characteristics of haemorrhoids have not been elucidated fully. Microscopically, haemorrhoidal piles are sinusoids (vascular structures without a muscular wall)^1^. Direct arteriovenous communications have been demonstrated histologically and radiologically, and some authors have noted a resemblance to erectile tissue^2^. Traditionally, haemorrhoidal piles frequently appear to be localized to the left lateral, right posterolateral and right anterolateral sites in the anal canal circumference with the patient in the lithotomy position; however, this configuration is demonstrated in less than 20 per cent of patients^3^. In reality, a wider network of arterial and venous vessels has been described^4^, although the distribution and relationship to rectal and anal layers is unclear.

Recently, haemorrhoidal disease (HD) has often been treated using non-excisional procedures. Some surgical techniques address the reduction of arterial inflow to haemorrhoids.

Transanal haemorrhoidal dearterialization (THD) and Doppler-guided haemorrhoidal artery ligation (DG-HAL) are the main surgical procedures with this aim, and use specifically designed devices for arterial ligation in the lower rectum guided by a Doppler signal^5^. Stapled haemorrhoidopexy (SH) divides the haemorrhoidal arteries in the suture line^6^.

Assessment of the optimal site for these surgical approaches should improve the clinical efficacy. The purpose of this study was to localize precisely the arteries running into the rectum and directed to haemorrhoids.

## Methods

The local institutional review board approved this study. Patients with HD were enrolled prospectively. Each patient signed an informed consent form regarding the procedures and purpose of the study. All patients had anal bleeding with or without haemorrhoidal prolapse. Before inclusion in the study, an accurate diagnostic assessment, including patient history, physical examination, anoproctoscopic and colonoscopic findings, if indicated, confirmed HD. Patients with chronic bowel inflammatory disease, anal fissures, anal fistulas or abscesses, and a history of pelvic surgery and/or radiotherapy were excluded.

**Fig. 1:**
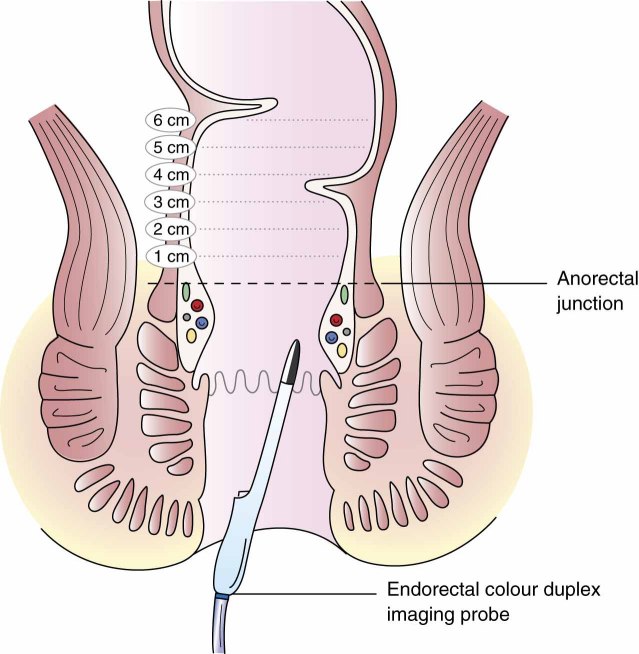
Topographic schematic diagram showing different levels of the colour duplex imaging examination

The enrolled patients underwent endoanal–endorectal ultrasonography (ERUS) and colour duplex imaging performed by a single operator. An ultrasound system (Pro-Focus Green™; BK Medical, Herlev, Denmark) fitted with endoanal–endorectal probes (models 2052 and 8848; BK Medical) was used. Before ultrasound examinations, the patients were prepared with two enemas to flush the rectum. During ERUS, the proximal edge of the puborectalis sling was identified to localize the anorectal junction (ARJ). The ARJ was regarded as the best reference point during anorectal ultrasonography. The anal dentate line cannot be identified using ultrasound techniques, and in patients with HD the anal pecten can frequently be displaced. The lower rectal circumference was subdivided into six sectors (left anterolateral, left lateral, left posterolateral, right posterolateral, right lateral and right anterolateral) (Fig. 1). From the upper limit (6 cm above the ARJ), the same procedure was repeated every 1 cm until the lower limit was reached (1 cm above the ARJ) (Fig. 1). A total of 300 sectors were studied for each of the six rectal levels.

Using combined colour duplex imaging, the courses of arteries that reached haemorrhoidal piles were followed carefully. All perirectal arteries that were not directed to haemorrhoids (vaginal, prostatic, and seminal vesicle arteries) were excluded from the study.

Arteries were classified according to their location in the rectal wall: running within the submucosa, between the submucosa and the rectal muscle, within the rectal muscle, between the rectal muscle and the perirectal fat, or outside the rectal wall. The distance between the centre of the arterial lumen and the ultrasound probe surface (defined as ‘arterial depth’) was calculated.

Close contact was maintained with the rectal mucosa, but care was taken to avoid applying excessive pressure to the rectal wall with the ultrasound probe to minimize any distortion of the ultrasonographic and Doppler signals owing to arterial occlusion or compression. For each sector investigated, at least one picture was obtained for review after the examination.

### Statistical analysis

The mean(s.d.) value was calculated for each recorded parameter. One-way ANOVA was used to compare means. The Bonferroni method was used for multiple comparisons, when appropriate. *P* < 0·050 was considered statistically significant.

## Results

Fifty patients (36 men, 14 women) with a mean(s.d.) age of 47·1(13·1) years were studied. Five patients (10 per cent) had grade II, 41 (82 per cent) had grade III and four (8 per cent) had grade IV haemorrhoids.

**Fig. 2:**
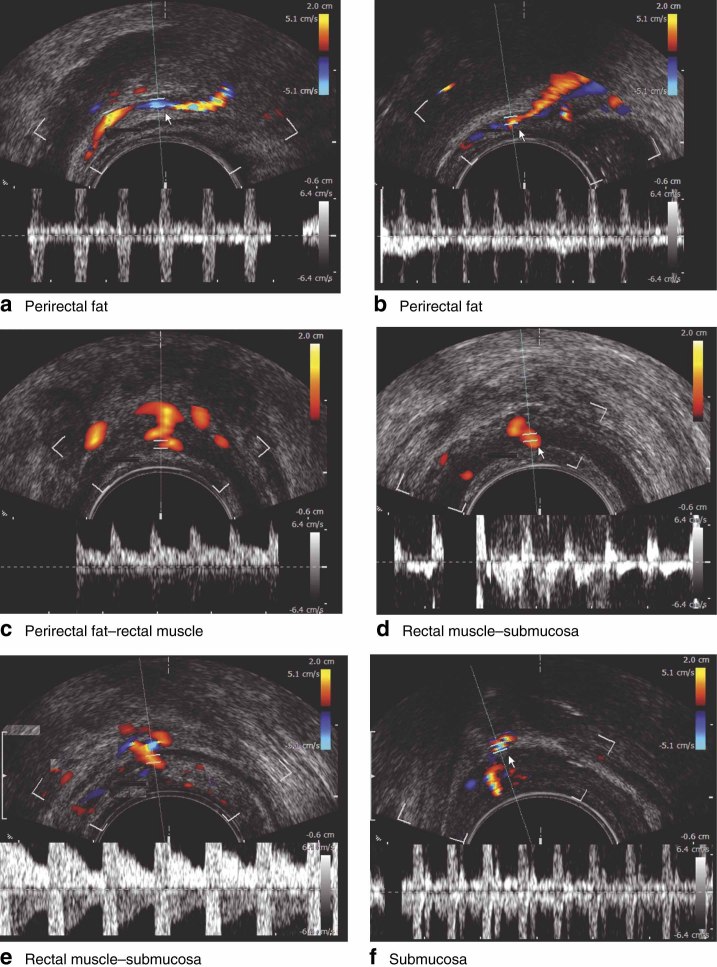
Colour duplex imaging examples of different locations of arteries in relation to the rectal wall: **a,b** perirectal fat; **c** perirectal fat–rectal muscle; **d,e** rectal muscle–submucosa; **e** submucosa

Significantly fewer sectors in the upper part of the low rectum had an arterial supply directed to the haemorrhoids than in the lower part (64·3, 66·0 and 66·0 per cent at 6, 5 and 4 cm above the ARJ respectively *versus* 98·3, 99·3 and 99·7 per cent at 3, 2 and 1 cm respectively; *P* < 0·001). Fig. 2 shows colour duplex imaging samples of different artery locations in relation to the rectal wall layers. The distribution of haemorrhoidal arteries in relation to rectal layers and distance from the ARJ is shown in Table 1. In the majority of the upper sectors (97·9 per cent at 6 cm and 90·9 per cent at 5 cm from the ARJ), haemorrhoidal arteries were located in the perirectal fat, and only occasionally within the bowel wall. At 4 cm above the ARJ, a greater number of sectors had arteries located in the rectal muscle. At 3 cm, the arteries were shown to run into the submucosa in the majority of sectors, whereas at 2 and 1 cm above the ARJ almost all of the arteries had a submucosal location (in 96·6 and 100 per cent of sectors respectively); the differences were statistically significant (*P* < 0·001).

**Table 1:** Distribution of detectable haemorrhoidal arteries in relation to rectal sectors and wall layers

	No. of rectal sectors						
		
	Distance from anorectal junction						
		
Haemorrhoidal artery location	6 cm	5 cm	4 cm	3 cm	2 cm	1 cm	P*
Perirectal fat	189 (97·9)	180 (90·9)	84 (42·4)	8 (2·7)	0 (0)	0 (0)	< 0·001
Perirectal fat–rectal muscle	0 (0)	5 (2·5)	48 (24·2)	9 (3·1)	0 (0)	0 (0)	< 0·001
Rectal muscle	4 (2·1)	9 (4·5)	34 (17·2)	23 (7·8)	5 (1·7)	0 (0)	< 0·001
Rectal muscle–submucosa	0 (0)	3 (1·5)	27 (13·6)	57 (19·3)	5 (1·7)	0 (0)	< 0·001
Submucosa	0 (0)	1 (0·5)	5 (2·5)	198 (67·1)	288 (96·6)	299 (100)	< 0·001

Values in parentheses are percentages.

* One-way ANOVA and Bonferroni tests.

No haemorrhoidal arteries were detected in the left and right anterolateral sectors at 6, 5 and 4 cm above the ARJ, whereas such arteries were identified in the other sectors. At the lower three levels (3, 2 and 1 cm above the ARJ), haemorrhoidal arteries were identified in nearly all circumferential sectors (Table 2). The mean haemorrhoidal arterial depth was significantly lower in more distal sectors than in more proximal sectors; a statistical comparison between each level showed all differences to be significant (*P* < 0·001) (Table 3). This was a consistent finding in each rectal sector (Table 4). When mean arterial depths at each rectal level were compared, the differences between sectors were not statistically different at 6 cm above (*P* = 0·674) or 1 cm below (*P* = 0·865) the ARJ, whereas differences between sectors were statistically significant at 5, 4, 3 and 2 cm above the ARJ (*P* = 0·022, *P* = 0·020, *P* < 0·001 and *P* = 0·005 respectively).

**Table 2:** Rectal sectors with detectable haemorrhoidal arteries in relation to distance from anorectal junction

	Distance from anorectal junction					
	
Rectal sector	6 cm	5 cm	4 cm	3 cm	2 cm	1 cm
Left anterolateral	0 (0)	0 (0)	0 (0)	47 (94)	50 (100)	50 (100)
Left lateral	47 (94)	49 (98)	49 (98)	50 (100)	50 (100)	50 (100)
Left posterolateral	49 (98)	50 (100)	50 (100)	50 (100)	50 (100)	50 (100)
Right posterolateral	48 (96)	49 (98)	49 (98)	49 (98)	49 (98)	49 (98)
Right lateral	49 (98)	50 (100)	50 (100)	50 (100)	50 (100)	50 (100)
Right anterolateral	0 (0)	0 (0)	0 (0)	49 (98)	49 (98)	50 (100)

Values in parentheses are percentages.

**Table 3:** Arterial depth in relation to level of rectal circumference

	Distance from anorectal junction						
		
	6 cm	5 cm	4 cm	3 cm	2 cm	1 cm	P*
Arterial depth (mm)	8·3(1·5)	6·6(1·4)	5·1(1·2)	3·3(0·9)	2·4(0·6)	1·9(0·4)	< 0·001

Values are mean(s.d.).

* One-way ANOVA and Bonferroni tests.

**Table 4:** Arterial depth in relation to level of rectal circumference and sector

	Arterial depth (mm)						
	
	Distance from anorectal junction						
	
Rectal sector	6 cm	5 cm	4 cm	3 cm	2 cm	1 cm	P*
Left anterolateral	—	—	—	2·6(0·7)	2·2(0·6)	1·9(0·5)	< 0·001
Left lateral	8·0(1·7)	6·1(1·4)	4·6(0·9)	3·4(0·7)	2·4(0·6)	1·9(0·4)	< 0·001
Left posterolateral	8·5(1·4)	7·0(1·4)	5·3(1·3)	3·5(0·9)	2·4(0·6)	1·9(0·4)	< 0·001
Right posterolateral	8·3(1·5)	6·7(1·5)	5·2(1·2)	3·6(0·8)	2·5(0·5)	2·0(0·4)	< 0·001
Right lateral	8·3(1·5)	6·7(1·3)	5·2(1·2)	3·6(1·0)	2·6(0·8)	1·9(0·4)	< 0·001
Right anterolateral	—	—	—	2·9(0·8)	2·2(0·5)	1·8(0·4)	< 0·001
P*	0·674	0·022	0·020	< 0·001	0·005	0·865	—

Values are mean(s.d.).

* One-way ANOVA and Bonferroni tests.

## Discussion

The pathogenesis of HD is unclear, but is probably multi-factorial. A number of elements have been claimed to be causative or predisposing factors. Disruption of supportive tissue surrounding haemorrhoids is considered to be an important factor in haemorrhoidal prolapse^7^ and a number of inflammatory mediators have also been cited^8^, ^9^. A hypertonic internal anal sphincter has frequently been associated with HD and is regarded as a possible cause of haemorrhoidal symptoms^10^. Haemorrhoidal vascularization appears to play a central role in the pathophysiology of HD.

Hyperplasia of the arteriovenous network within the anorectal submucosa (corpus cavernosum recti, CCR) results in increased vascular pressure. Blood overflow to the CCR should also cause increased intravascular pressure, and is thus a significant predisposing factor for HD^11^. Aigner and colleagues^12^ confirmed the relationship between arterial overflow and HD. Using a transperineal Doppler probe to investigate haemorrhoidal arteries, they found a significantly higher arterial calibre and flow velocity in patients with HD compared with controls. They then hypothesized that the coordinated filling and drainage of the anorectal vascular plexus is regulated by the intrinsic vascular sphincter mechanism, and that the morphological and functional failure of this vascular system may contribute to the development of HD^13^.

A comprehensive understanding of anorectal vascularization should contribute to outlining the pathophysiology of HD. A recent study by Schuurman and co-workers^14^ highlighted how vascularization of the CCR is provided almost exclusively by branches of the superior rectal artery (SRA), a terminal branch of the inferior mesenteric artery. A previous study by Shafik and Mostafa^15^ indicated that the lower half of the rectum is vascularized by the terminal branches of the SRA (two or three main branches), with plexiform patterns at the ends. The middle rectal artery has been reported in only 50 per cent of cadaver specimens^15^, and the functional role of this artery seems negligible in light of these anatomical inconsistencies. DiDio and colleagues^16^ also studied the middle rectal artery in 30 cadavers; it was present in 56·7 per cent of specimens, bilaterally (36·7 per cent) or unilaterally (20·0 per cent). The middle rectal artery arose from the internal pudendal artery in 40 per cent of specimens, the inferior gluteal artery in 26·7 per cent, and the internal iliac artery in 16·8 per cent. The consistent findings of the above studies appear to demonstrate that the SRA branches play a predominant role in CCR vascularization. Therefore, it is particularly important to define the topography of these vessels within the rectal–perirectal area. Aigner *et al.*^17^ analysed five macroscopic preparations of human pelvis; they described the division of the SRA into left and right branches, then into three to five terminal branches penetrating the rectal wall in the middle and lower rectum. On examining microscopic preparations from 27 fetuses, they identified two to four terminal vessels penetrating the rectal wall and reaching the submucosa, especially in the posterolateral position (71 per cent of specimens)^17^.

In the present study, the majority of arterial branches at the three highest levels (6, 5 and 4 cm from the ARJ) were located outside the rectal wall in the right lateral, right posterolateral, left posterolateral and left lateral sectors, where these vessels arise. In contrast, no haemorrhoidal arteries were detected in several sectors in the higher three levels; in particular, none was found in the right and left anterolateral sectors. These findings suggest that the arterial pulses detected by Doppler ultrasonography in the anterior highest three levels of the low rectum during surgical procedures using this technology can be regarded as being generated by vessels that are not directed to haemorrhoids. In contrast, in the lower 2 cm, haemorrhoidal arteries were detected in 98 per cent of sectors; specifically, at 2 and 1 cm from the ARJ, arteries were identified in the submucosa in 96·6 per cent and 100 per cent of sectors respectively. These features can be confirmed easily during Doppler-guided surgical procedures. Investigation of the position of the arteries in relation to rectal layers and levels showed that the mean arterial depth decreased significantly from the highest to the lowest level, reaching the shallowest depth at the most distal 2 cm of the rectum where nearly all of the arteries were in the submucosa; this feature was invariably found regardless of the circumferential sector investigated.

Both anatomical and physiological evidence obtained from the literature and the present study has implications for the various therapeutic approaches that are currently available. In this regard, the most innovative surgical techniques are SH (also known as Longo's technique) and Doppler-guided ligation of haemorrhoidal arteries, including THD and DG-HAL techniques. The goal of the first method is to treat haemorrhoidal prolapse by resecting the rectal mucosa approximately 3–4 cm above the dentate line^18^; however, the level of anastomosis is frequently unpredictable as it is affected by the traction applied to the previously performed rectal purse-string. In fact, it has been established that, even though SH is performed according to well established technical guidelines, the intended location of the staple line is too difficult to standardize, as demonstrated by the wide range of anatomicopathological results reported by Ohana and co-workers^19^. Based on these data and the present findings, only a suture located within the distal 2 cm of the low rectum plays a role in the control of arterial overflow in patients treated with SH. However, even if SH and Doppler-guided ligation of haemorrhoidal arteries were applied, some doubts about Longo's procedure remain with respect to the circumferential suture performed with the specifically designed stapling device. This type of suture cannot ensure selective ligation of haemorrhoidal arteries as it can also involve both major and minor arterial vessels. Moreover, the circumferential suture could generate an unpredictable risk of venous outflow blockage, thus damaging the drainage system, as described previously^13^.

The correlation between SH and rectal vascularization was highlighted in another study in which a perineal Doppler probe was used in patients who underwent SH for HD and a group of healthy subjects^20^. Baseline measurements differed significantly between patient and control groups. Postoperative follow-up showed no significant alterations in physiological parameters. Patients with a higher rate of recurrence of HD had higher baseline arterial flow velocity values. The study showed that SH did not reduce arterial inflow in the vessels feeding the anorectal vascular plexus. The present data may explain the reasons for the failure to reduce vascular overflow. In that study^20^, the anastomosis was performed 3·5–4 cm above the dentate line, a level at which most terminal arterial branches are not in the submucosa. Indeed, a meta-analysis of large-scale studies of patients undergoing SH demonstrated that these patients are more likely to develop recurrent HD with prolapse and bleeding at any time than those having conventional haemorrhoidectomy^21^, ^22^.

The goal of THD and DG-HAL is significantly to reduce arterial overflow to haemorrhoidal piles by dearterialization, that is Doppler-guided ligation of the haemorrhoidal arteries in the upper part of the low rectum. The results of these operations seem promising^23^–^31^. In particular, most studies have shown that recurrent bleeding is limited to a minority of patients (5–20 per cent after THD; 1–21 per cent after DG-HAL)^23^–^31^.

However, traditional dearterialization might fail to include the haemorrhoidal arteries in some sites owing to their deep location (within the muscularis propria or in perirectal fat), particularly on the anterior side of the rectum. The reported frequency of recurrent bleeding in patients undergoing dearterialization alone using the ‘high arterial ligation’ technique (31 per cent)^26^ supports this view. When mucopexy is included in THD or DG-HAL procedures, the possibility of excluding arteries may be lower as the running suture (even one that begins in the upper part of the low rectum to perform high ligation of haemorrhoidal arteries) is usually continued by transfixing the mucosa and submucosa to the ARJ, thus involving arterial branches directed to the haemorrhoidal piles.

By selectively ligating the haemorrhoidal arteries using a very precise Doppler system^26^, the THD technique can accurately identify the location of arterial vessels in the submucosa of the low rectum, thus achieving a significant reduction in arterial overflow to haemorrhoids. Based on the present findings, dearterialization should be more effective if performed 1 and 2 cm from the ARJ, where almost all of the arteries are localized in the submucosa, with a mean depth of 1·9–2·4 mm. In contrast, arterial ligation 3 cm from the ARJ may not be effective in certain sectors. At this level, 67·1 per cent of the vessels identified were located in the submucosa. Above this level, a smaller percentage of submucosal arteries was found, possibly making dearterialization less accurate at higher levels.

This study provides new insight into the functional anatomy of haemorrhoids, with a direct impact on pathophysiology and treatment. The location of almost all the branches of the SRA in the submucosa provides a clear target for surgical treatments that recognize vascular overflow as a fundamental factor in the aetiology of HD. Optimizing dearterialization is essential for improving clinical outcome. In this regard, Doppler imaging plays a pivotal role during the surgical procedure by providing precise identification, then guided ligation of arteries. Clinical trials are required to confirm the therapeutic implications of these findings.
